# Single-incision laparoscopic total extraperitoneal repair for a Grynfeltt hernia: a case report

**DOI:** 10.1186/1752-1947-8-16

**Published:** 2014-01-15

**Authors:** Ching-Ting Wei, Yaw-Sen Chen, Cheuk-Kwan Sun, Kun-Chou Hsieh

**Affiliations:** 1 Division of General Surgery, Department of Surgery, I-Shou University, No.1, Yida Road, Jiao-su Village, Yan-chao District, Kaohsiung City 824, Taiwan; 2 Department of Emergency Medicine, E-Da Hospital, I-Shou University, No.1, Yida Road, Jiao-su Village, Yan-chao District, Kaohsiung City 824, Taiwan

**Keywords:** Extraperitoneal, Grynfeltt hernia, Single-incision laparoscopy

## Abstract

**Introduction:**

A superior lumbar hernia, which is also known as a Grynfeltt hernia, is a rare abdominal wall defect that can be primary or secondary to trauma or orthopedic surgery. The anatomic location of a lumbar hernia makes diagnosis and repair challenging. We successfully repaired a lumbar hernia using a single-incision laparoscopic total extraperitoneal approach. To the best of our knowledge, this is the first report of the use of this surgical technique in the treatment of a primary Grynfeltt hernia.

**Case presentation:**

A 76-year-old Taiwanese man presented to our hospital with a left lower bulging mass noted for over three months. Abdominal computed tomography revealed a left Grynfeltt hernia. We performed a single-incision laparoscopic total extraperitoneal repair. Our patient was discharged uneventfully on the fourth day after the operation. There was no evidence of recurrence after six months of follow-up.

**Conclusion:**

A laparoscopic total extraperitoneal repair for a lumbar hernia provides an excellent operative view and minimal invasiveness. The single-incision technique also provides better cosmetic outcomes. Our experience suggests that the single-incision laparoscopic total extraperitoneal approach may be a feasible and safe alterative to conventional approaches in lumbar hernia repair.

## Introduction

A lumbar hernia is a rare type of abdominal wall defect over the lumbar triangles that can be categorized into superior (Grynfeltt) and inferior (Petit) by anatomic location. The four borders of Grynfeltt or superior lumbar hernia are the 12th rib superiorly, quadratus lumborum muscle medially, iliac crest inferiorly, and internal oblique muscle laterally. The external oblique and latissimus dorsi muscles form its roof, and its floor comprises the transversalis fascia and the aponeurosis of the transversus abdominis muscle. Because of the anatomic location, the diagnosis and repair of a lumbar hernia are challenging. With the improvements in laparoscopic techniques, minimally invasive surgery has emerged as an increasingly popular therapeutic option in hernia repair in comparison to the conventional open approach. To the best of our knowledge, this report is the first description in the world of the use of single-incision laparoscopic total extraperitoneal approach in the repair of primary Grynfeltt hernia.

## Case presentation

**Figure 1:**
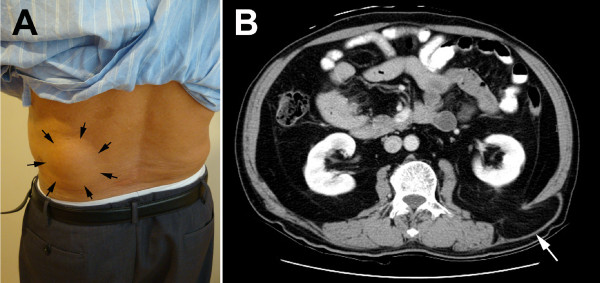
**Physical examination and computed tomography findings on admission. (A)** Left lower back bulging mass of size 10cm × 6cm (arrows). **(B)** Abdominal computed tomography demonstrating retroperitoneal fat protruding through the superior lumbar triangle (arrow).

A 76-year-old Taiwanese man presented to our hospital with a left lower bulging mass noted for over three months. He had no known history of systemic diseases, previous abdominal trauma or surgery. A physical examination showed a 10cm × 6cm reducible soft mass in his left lower flank area (Figure 1A). No tenderness or numbness was noted. Abdominal computed tomography revealed a herniation of abdominal fat, protruding through the superior lumbar triangle defined by his quadratus lumborum muscle, 12th rib and internal oblique muscle (Figure 1B). We admitted our patient for elective surgery with the diagnosis of lumbar hernia.

**Figure 2:**
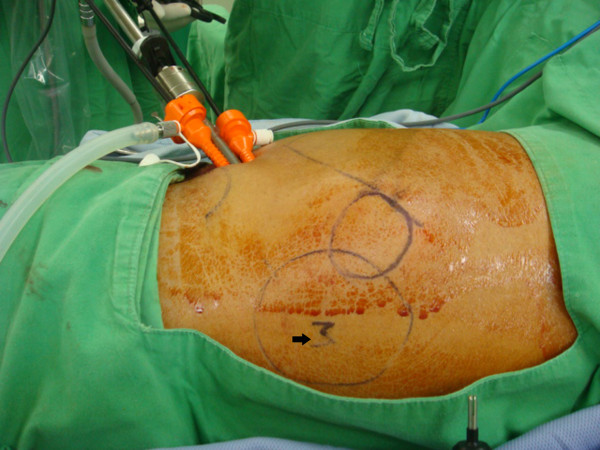
Position of three trocars over the anterior axillary line directed toward the bulging mass (M).

**Figure 3:**
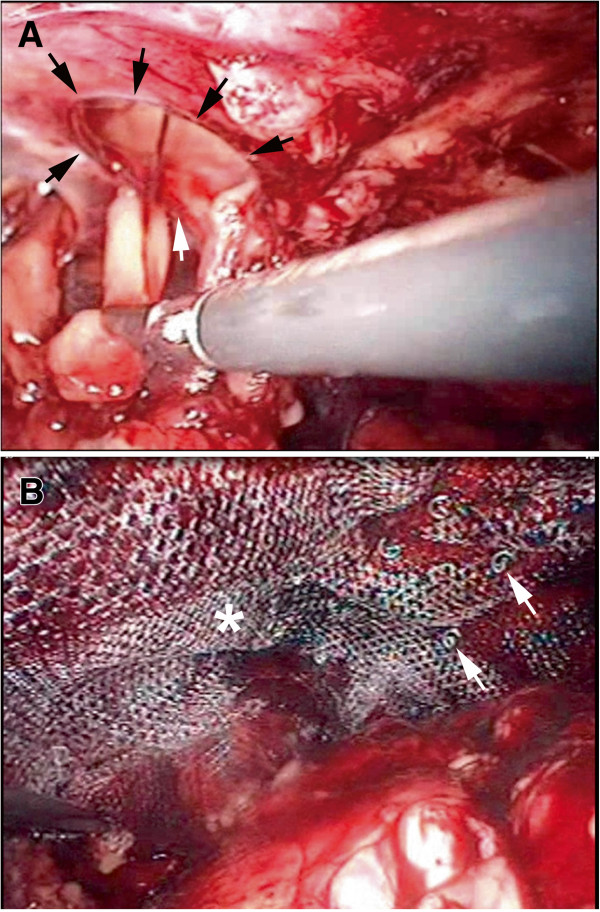
**Intraoperative images of the hernia repair. (A)** Detachment of extraperitoneal adipose tissue and exposure of the fascia defect (black arrows) at the initial stage of hernia repair. **(B)** Securing of polypropylene mesh (arrows) after complete patching of the fascia defect (asterisk).

We placed our patient in a right decubitus position, and made a 3cm transverse incision over the left anterior axillary line between his 12th rib and iliac crest. We dissected the muscular fascia layer towards the preoperative mark of the bulging mass using the blunt finger technique, then inserted a 10mm trocar through the incision. Under the guidance of a 10mm laparoscopic camera, we placed two 5mm working trocars into different sites of the fascia layer (Figure 2). The wound was covered with Vaseline gauze to maintain adequate extra-peritoneal pressure for satisfactory exposure of the surgical field. We carefully dissected the extraperitoneal space by detaching extraperitoneal adipose tissue from the hernia defect (Figure 3A). The hernia defect was found to be 3cm × 3cm. After dissecting the surrounding soft tissue to a minimum of 5cm from the hernia defect, we trimmed a 13cm × 13cm polypropylene mesh to a shape similar to that of the fascia defect and inserted this via a 10mm trocar. Finally, we secured the mesh with autosuture stapler (ProTack 5mm, Covidien, Mansfield MA, USA) to prevent migration (Figure 3B).

**Figure 4:**
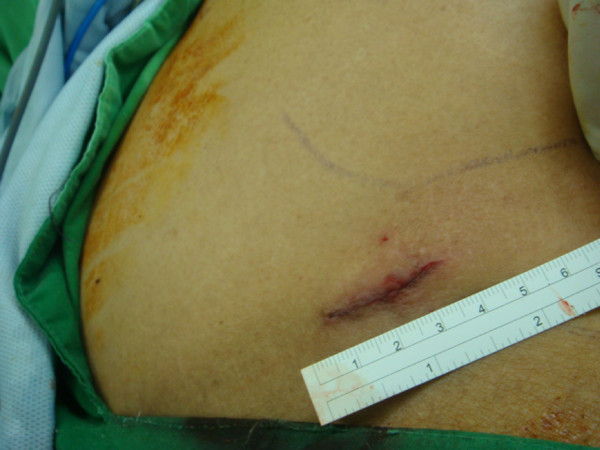
Postoperative wound over left flank after application of the single-incision technique.

Our patient recovered satisfactorily from general anesthesia with a single wound over his left flank (Figure 4). Postoperative complications including seroma or wound infection were not noted. Our patient was discharged uneventfully on the fourth day after the operation. He returned to the clinic 18 months later without evidence of recurrence.

## Discussion

Lumbar hernias account for only 2% of all abdominal hernias [1]. There are only about 300 cases reported in the English literature since its first description by Barbette in 1672. The anatomy of the inferior and superior lumbar triangle was reported by Petit and Grynfeltt in 1783 and 1866, respectively [2]. Lumbar hernias can be categorized into congenital (20%) or acquired, which have been further classified into two types: primary (55%) and secondary (25%). Whereas primary lumbar hernias have no apparent provoking cause, the etiologies of secondary lumbar hernias include trauma, infection, orthopedic surgery and kidney surgery. A superior lumbar hernia, which is also known as a Grynfeltt hernia, is a primary lumbar hernia that is more common, larger and deeper compared to the inferior lumbar hernia [3].

Lumbar hernias mostly present as an asymptomatic protruding mass over the lower back or flank area. Differential diagnoses including lipoma, local abscess and postoperative hematoma; even neoplasms should first be ruled out. The diagnosis is based on the clinical history and physical findings. Computed tomography is a good tool not only for definite diagnosis but also for preoperative planning [4,5].

Currently, there are three approaches to lumber hernia repair: open, intraperitoneal and extraperitoneal laparoscopic. Open primary repair for a lumbar hernia was first described by Owen in 1888, followed by closure of the fascia defect with the tensor fascia latae flap by Dowd in 1907 [6] and repair using prosthetic mesh by Alves *et al*. in 1996 [7]. The defect may be closed directly for smaller lumbar hernias, whereas a muscular flap or prosthetic mesh should be considered for achieving tension-free closure in the management of larger hernia defects. A laparoscopic intraperitoneal approach for traumatic lumbar hernias was first reported by Burick and Parascandola in 1996, and for a primary lumbar hernia by Heniford *et al*. in 1997 [2,8]. This intraperitoneal access provides more accurate localization, better fascia reconstruction, less wound pain, and superior cosmetic results compared to the open approach [9]. However, intraperitoneal access with manipulation of intra-abdominal organs may contribute to subsequent intra-abdominal adhesions. Laparoscopic extraperitoneal repair, the most recently developed technique first described by Meinke in 2003 [10], not only allows precise anatomic localization of the hernia defect and minimal wound discomfort [11], but also avoids unnecessary manipulation of visceral organs and reduces the possibility of subsequent intra-abdominal adhesions. Therefore, it may be the ideal method for lumbar hernia repair.

In our case, the right decubitus position was adopted because of the protruding mass. The reason for making the incision over the left anterior axillary line between our patient’s 12th rib and iliac crest was to facilitate instrument manipulation for the management of the prolapsed mass and also for hernia repair. Due to his poor self-care ability and intolerance to wound pain, our patient’s hospital stay was longer than average (one to two days after the surgery).

## Conclusion

Compared with the open and intraperitoneal laparoscopic approaches, laparoscopic total extraperitoneal repair for a lumbar hernia provides an excellent operative view and minimal invasiveness. The single-incision technique also provides better cosmetic outcomes. Our experience suggests that the single-incision laparoscopic total extraperitoneal approach may be a feasible and safe alternative to the conventional approaches in lumbar hernia repair.

## Consent

Written informed consent was obtained from the patient for publication of this case report and accompanying images. A copy of the written consent is available for review by the Editor-in-Chief of this journal.

## Competing interests

The authors declare that they have no competing interests.

## Authors’ contributions

CTW carried out collection and assembly of data, data analysis and interpretation, and manuscript writing. YSC provided administrative support. CKS contributed to the manuscript writing. KCH was responsible for the conception and design, provision of study material or patients, collection and assembly of data, data analysis and interpretation, and final approval of the manuscript. All authors read and approved the final manuscript.
